# GapB Is Involved in Biofilm Formation Dependent on LrgAB but Not the SinI/R System in *Bacillus cereus* 0-9

**DOI:** 10.3389/fmicb.2020.591926

**Published:** 2020-12-07

**Authors:** Juanmei Zhang, Li Meng, Yubing Zhang, Lidan Sang, Qing Liu, Linlin Zhao, Fengying Liu, Gang Wang

**Affiliations:** ^1^ Engineering Research Center for Applied Microbiology of Henan Province, Kaifeng, China; ^2^ School of Pharmaceutical, Henan University, Kaifeng, China; ^3^ Institute of Microbial Engineering, Laboratory of Bioresource and Applied Microbiology, School of Life Sciences, Henan University, Kaifeng, China

**Keywords:** biofilm, glyceraldehyde-3-phosphate dehydrogenase, extracellular DNA, gluconeogenesis, GapB

## Abstract

*Bacillus cereus* 0-9, a Gram-positive endospore-forming bacterium isolated from healthy wheat roots, has biological control capacity against several soil-borne plant diseases of wheat such as sharp eyespot and take-all. The bacterium can produce various biofilms that differ in their architecture and formation mechanisms, possibly for adapting to different environments. The *gapB* gene, encoding a glyceraldehyde-3-phosphate dehydrogenase (GAPDH), plays a key role in *B. cereus* 0-9 biofilm formation. We studied the function of GapB and the mechanism of its involvement in regulating *B. cereus* 0-9 biofilm formation. GapB has GAPDH activities for both NAD^+^- and NADP^+^-dependent dehydrogenases and is a key enzyme in gluconeogenesis. Biofilm yield of the Δ*gapB* strain decreased by 78.5% compared with that of wild-type *B. cereus* 0-9 in lysogeny broth supplemented with some mineral salts (LBS), and the Δ*gapB*::*gapB* mutants were recovered with *gapB* gene supplementation. Interestingly, supplementing the LBS medium with 0.1–0.5% glycerol restored the biofilm formation capacity of the Δ*gapB* mutants. Therefore, GapB regulates biofilm formation relative to its function in gluconeogenesis. To illustrate how GapB is involved in regulating biofilm formation through gluconeogenesis, we carried out further research. The results indicate that the GapB regulated the *B. cereus* 0-9 biofilm formation independently of the exopolysaccharides and regulatory proteins in the typical SinI/R system, likely owing to the release of extracellular DNA in the matrix. Transcriptome analysis showed that the *gapB* deletion caused changes in the expression levels of only 18 genes, among which, *lrgAB* was the most significantly increased by 6.17-fold. We confirmed this hypothesis by counting the dead and living cells in the biofilms and found the number of living cells in the biofilm formed by the Δ*gapB* strain was nearly 7.5 times than that of wild-type *B. cereus* 0-9. Therefore, we concluded that the GapB is involved in the extracellular DNA release and biofilm formation by regulating the expression or activities of LrgAB. These results provide a new insight into the regulatory mechanism of bacterial biofilm formation and a new foundation for further studying the stress resistance of *B. cereus*.

## Introduction

Microorganisms perform coordinated activities were previously thought to be restricted to multicellular organisms (Song et al., 2014; Yan et al., 2016; Wan et al., 2018). Bacteria exhibit many social activities and display an array of contact-dependent interaction systems, which have evolved to facilitate direct cell-to-cell communication (Li et al., 2018). Biofilm formation, a typical behavior representing part of the bacterial social repertoire, is a constructive survival strategy that enables bacteria to use a greater variety of nutrients, endure rapid environmental changes, and resist multiple adverse threats (Moons et al., 2009). *Bacillus cereus* produces various biofilms that differ in their architecture and formation mechanisms, possibly to adapt to various environments (Majed et al., 2016).

The complex architecture of a biofilm is maintained by an extracellular matrix of exopolysaccharides, proteins, and DNA (Candela et al., 2019). Extracellular polysaccharide (EPS), produced by a biofilm community, form the microenvironment for cells in the biofilm (Flemming et al., 2007). The EPS matrix is well hydrated and plays various roles, including adhering the biofilm to surfaces, sequestering substances from the environment, and protecting the bacteria from predators (Vilain et al., 2009). Biofilm formation requires a complex regulatory pathway that coordinates gene expression with external environmental conditions and structural components involved in assembling a protective extracellular matrix (Romero, 2013; Vlamakis et al., 2013). Controlling biofilm formation depends on a molecular switch that comprises two transcription repressors and two anti-repressors (Diethmaier et al., 2014). Central to this regulatory switch is the repressor, SinR (Newman et al., 2013), a DNA-binding protein, that represses transcription of operons involved in producing both an exopolysaccharide (Branda et al., 2001) and the protein component of the matrix. SinR activity is controlled by the relative levels of various antagonists, the foremost of which is SinI (Kearns et al., 2005), which causes de-repression by binding to SinR, so that the SinI-SinR complex can no longer bind to DNA (Lewis et al., 1996, 1998). The EPS of *P. aeruginosa* biofilms contains DNA; therefore, young biofilms can be dislodged using DNase (Whitchurch et al., 2002). Nucleic acid in the EPS matrix of the biofilms has been termed as extracellular DNA and is required for the structural integrity of the biofilms of various Gram-negative and Gram-positive bacteria, including *B. cereus* (Vilain et al., 2009). Southern blotting revealed that an extracellular DNA from biofilms was similar to chromosomal DNA. At present, *B. cereus* is not known to take up DNA *via* natural methods, nor are there any reported indications of a DNA export system in its genome sequence. Thus, it remains unclear how this extracellular DNA is exported and involved in *B. cereus* biofilm formation.

GAPDH is a key glycolytic enzyme whose primary function is to phosphorylate glyceraldehyde-3-phosphate (G-3-P) to 1,3-diphosphoglycerate during glucose metabolism (Purves et al., 2010). This enzyme is considered as a housekeeping protein that present in nearly all organisms, where it performs the metabolic functions essential for survival (Giménez et al., 2014). Although GAPDH plays an essential role in energy production, it has more functions than those of a classic glycolytic protein. Several studies have described GAPDH as a multifunctional protein in numerous biological processes in pathogens, probiotics, mammals, and plants (Arutyunova et al., 2013). The multifunctionality of intracellular GAPDH has been widely studied in humans; however, these studies are scarce in bacteria. Recent reports on *Streptococcus pyogenes* and *Escherichia coli* evidenced that the bacterial GAPDH may also be involved in intracellular processes such as transcriptional regulation, DNA repair, and quorum sensing signaling (Giménez et al., 2014). Recent research reports that the different concentrations of glucose can change the structural characteristics of *Candida albicans* biofilms (Figueira et al., 2020). And before that, the effect of glycolysis on bacterial biofilm formation has been rarely reported.

*Bacillus cereus* 0-9, a gram-positive endospore-forming bacterium, isolated from healthy wheat roots, has biological control capacity against several soil-borne plant diseases of wheat such as sharp eyespot and take-all. Biofilm formation is crucial for this bacterium to use variety of nutrients, endure rapid environmental changes, and resist multiple adverse threats. In this work, we uncovered an important role for GapB, one of GAPDHs, in the formation of biofilms *in vitro*. We previously sequenced and annotated the whole genome of *B. cereus* 0-9 and found that three *gap* genes encode GAPDH: *gapA*, *gapB*, and *gapN*. It has been reported that different GAPDH have different functions in glycometabolism (Purves et al., 2010; Romero et al., 2014). For example, GapA is expressed to perform glycolytic function in the presence of glucose, while gluconeogenesis requires GapB to be expressed in the absence of glucose. But the function of GapN is unclear now. The function of GAPDH in glucose metabolism pathways has been studied deeply. However, to our knowledge, the role of this class of enzymes during biofilm formation in *B. cereus* 0-9 was unnoticed. Furthermore, we also found wild-type *B. cereus* 0-9 can form architecturally complex colonies and pellicles under laboratory conditions (Zhang et al., 2020a). Studying the genes related to this biofilm formation will enable better understanding the basic characteristics of biocontrol strains. To our knowledge, the function and roles of GAPDH in glucose metabolism pathway has been studied deeply; however, how GAPDH affects *B. cereus* biofilm formation is unclear. Hence, we studied the biofilm productions of Δ*gap* strains and investigated the mechanism by which GAPDH regulates *B. cereus* 0-9 biofilm formation in this study. And our results strongly suggest that the molecular basis for GapB-dependent biofilm formation involves control over the autolysis regulator system LrgAB and most likely eDNA release.

## Materials and Methods

### Strains, Plasmids, Culture Media, and Growth Conditions

The *B. cereus* 0-9 properties and culture conditions have been reported previously (Zhang et al., 2020b). Both *E. coli* 116 (*pir*+) and *E. coli* GM2163 (dam-) were incubated in lysogeny broth (LB) at 37°C overnight and used for plasmid propagation and demethylation, respectively. The plasmid, pAD (Takara, Dalian, China), containing ampicillin- and erythromycin-resistance genes, was used to develop a new fusion vector, pAD123-Pgal, which was used to generate deletions in *B. cereus* open reading frames (Arnaud et al., 2004). The plasmid, pMAD (Miaoling, Wuhan, China), containing ampicillin- and erythromycin-resistance genes and a heat-resistant *bgaB* gene, was used to construct gene deletion mutants. The 19 T-vector (Takara, Dalian, China), containing ampicillin-resistance genes, efficiently clones DNA fragments and was used to generate T-vector cloning. All tested strains and plasmids used in this experiment are listed in Table 1.

**Table 1:** Tested strains and plasmids used in this experiment.

Name	Properties and application	Source
Strains		
*B. cereus* 0-9	Wild type strain in this study	Kept in our laboratory, isolated from wheat root.
*E. coli* 116 (*pir*+)	Plasmid propagation	Purchased from BioVector NTCC
*E. coli* GM2163 (dam-)	Demethylation	Purchased from BioVector NTCC
*E. coli* BL21	Protein expression	Purchased from BioVector NTCC
SL1001	The *gapA* gene deletion mutant, ∆*gapA*	Construct in this study
SL1002	The *gapA* gene deletion mutant, ∆*gapB*	Construct in this study
SL1003	The *gapA* gene deletion mutant, ∆*gapN*	Construct in this study
FPU1061I	The *sinI* gene defect mutant, ∆*sinI*	Construct in this study
FPU1062R	The *sinR* gene defect mutant, ∆*sinR*	Construct in this study
SL2001	The double knockout strain of *gapB* and *sinI* genes, ∆*gapB*∆*sinI*	Construct in this study
SL2002	The double knockout strain of *gapB* and *sinR* genes, ∆*gapB*∆*sinR*	Construct in this study
SL1004	The *glk* gene defect mutant, ∆*glk*	Construct in this study
SL2003	The double knockout strain of *glk* and *gapB* genes, ∆*gapB*∆*glk*	Construct in this study
SL7110	Δ*gapB* supplemented with *gapB* gene by allelic exchange, ∆*gapB*::*gapB*	Construct in this study
SL7120	Δ*gapB* supplemented with *gapB* gene by reverse compliment, ∆*gapB*/*gapB*_pro_	Construct in this study
SL7212	Δ*gapB*∆*sinR* mutant that supplemented with *gapB* gene, ∆*gapB*∆*sinR*::*gapB*	Construct in this study
SL7252R	Δ*gapB*∆*sinR* mutant that supplemented with *sinR* gene, ∆*gapB*∆*sinR*::*sinR*	Construct in this study
SL7211	Δ*gapB*∆*sinI* mutant that supplemented with *gapB* gene, ∆*gapB*∆*sinI*::*gapB*	Construct in this study
SL7251I	Δ*gapB*∆*sinI* mutant that supplemented with *sinI* gene, ∆*gapB*∆*sinI*::*sinI*	Construct in this study
ZL2011	The *lrgAB* gene defect mutant, ∆*lrgAB*	Construct in this study
ZL3011	The double knockout strain of *lrgAB* and genes, ∆*gapB*∆*lrgAB*	Construct in this study
Plasmid		
pAD	For gene knockout	Takara, Dalian
pMAD	For gene knockout	Miaoling, Wuhan
*gapA*-pMAD	For gene knockout, Amp^+^; Erm^+^	Construct in this study
*gapB*-pMAD	For gene knockout, Amp^+^; Erm^+^	Construct in this study
*gapN*-pMAD	For gene knockout, Amp^+^; Erm^++^	Construct in this study
*glk*-pMAD	For gene knockout, Amp^+^; Erm^++^	Construct in this study
pAD-pgal-JT	For reverse complementation	Stored in our laboratory
pAD-pgal-JT-*gapB*com	For gene complementation, Amp^+^; Cm^+^	Construct in this study
pAD-pgal-JT-*sinI*com	For gene complementation, Amp^+^; Cm^+ +^	Construct in this study
pAD-pgal-JT-*sinR*com	For gene complementation, Amp^+^; Cm^+^	Construct in this study
pMAD-*chi*	For gene complementation, Cm^+^	Construct in this study
pET28a*-gapB*	Expression of protein, Km^+^	Construct in this study

Biofilm formed by the tested strains was cultured in LBS and King broth medium supplemented with glucose (KBMS). In LBS medium, per 1,000 ml culture containing 10 g pryptone, 5.0 g yeast extract, 5.0 g NaCl, 1.0 g sodium citrate, 2.0 g (NH_3_)_2_SO_4_, 0.2 g MgSO_4_·H_2_O 14 g K_2_HPO_4_, 6.0 g KH_2_PO_4_, and 1.0 g glucose. In KBMS medium, per 1,000 ml culture containing 10 g pryptone, 10 g acid hydrolyzed casein, 1.5 g K_2_HPO_4_, 10 g glycerinum, 1.5 g MgSO_4_·H_2_O, and 10 g glucose, pH 7.4. Use of different carbon sources by the tested strains was determined in minimum energy require source (M-eps) medium, with 1,000-ml cultures containing 7.0 g K_2_HPO_4_, 3.0 g KH_2_PO_4_, 0.1 g MgSO_4_, 0.01 g CaCl_2_, 0.001 g FeSO_4_, 0.1 g NaCl, and 5.0 g acid hydrolyzed casein. Nutrient agar (NA) medium was used to observe the colony morphology of the tested strains with 1,000 ml of distilled water containing 5.0 g beef extract, 10 g peptone, 5.0 g NaCl, and 15 g agar powder.

### Construction of a *gapB* Gene Complementation Vector

To generate the gene complementation strains, pAD123-Pgal, a genetic reverse complementary system, was constructed using the pAD plasmid from the Bacillus Genetic Stock Center (Accession: ECE165; Columbus, OH, Unites States). Supplementary Figure S1 shows the structural map of the pAD123-Pgal vector. A DNA fragment containing the *B. cereus* 0-9 *gapB*-coding region and its native promoter was cloned into pAD123-Pgal at the *Mlu*I and *Xho*I sites to construct the recombinant plasmid, pAD123-Pgal*-gapB*_pro_, which was used to construct the complementation strains by electric shock conversion. Another vector, pMADchi, was constructed using the plasmid pMAD, with a combination of DNA fragments from upstream and downstream of the *chi* gene from *B. cereus* 0-9 inserted at the *EcoR*I and *Xho*I sites, and several additional restriction sites were inserted. Supplementary Figure S2 shows the structural map of the pMADchi plasmids. DNA fragment containing *gapB* gene from *B. cereus* 0-9 was cloned into pMADchi at the *BamH*I and *Xho*I sites, to construct the recombinant plasmid, pMADchi*-gapB*_orf_, which were used to construct the complementary strain by allelic exchange. The DNA fragment containing *sinI* or *sinR* gene from *B. cereus* 0-9 was cloned into pMADchi at the *Xho*I and *Eco*RI sites, or *Eco*RI and *BamH*I sites, respectively, to construct recombinant plasmid pMADchi*-sinI* and pMADchi*-sinR*.

### Construction of the Gene Mutant Strains and Complementation Experiments

Whole-genome sequencing results (GenBank: CP042874.1) and subsequent gene function annotation showed that the *B. cereus* 0-9 genome encodes three Gap proteins: GapA (Protein ID: QEF19539.1), GapB (Protein ID: QEF19056.1), and GapN (Protein ID: QEF15645.1). To investigate the functions of Gap in *B. cereus* 0-9 biofilm formation, we constructed the *gap*-knockout mutants, ∆*gapA*, ∆*gapB*, and ∆*gapN* by a previously described allelic exchange method (Xu et al., 2014; Zhang et al., 2020b). In detail, two fragments suitable for allelic exchange were created by cloning two *BamH*I-*Xho*I DNA fragments containing locus-specific flanking regions into the *BamH*I site of pMAD. These fragments were created *via* PCR using the primers in Supplementary Table S1. Ligation of the two fragments led to precise deletion of the respective open reading frame from the start to the stop codons and to generation of an *Xho*I site at the locus site. The resulting constructs were also used to create ∆*sinI*, ∆*sinR*, ∆*gapB*∆*sinI*, ∆*gapB*∆*sinR*, ∆*glk*, ∆*glk*∆*gapB*, ∆*lrgAB*, and ∆*lrgAB*∆*gapB* mutants of *B. cereus* 0-9.

The recombinant plasmid, pAD123-Pgal*-gapB*_pro_ was transformed into the competent cells of ∆*gapB* mutants to construct the reverse complimentary strain, ∆*gapB*::*gapB_pro_*. The other recombinant vectors, pMADchi-*gapB*_orf_, pMADchi-*sinR*, and pMADchi-*sinI* were transformed into the competent cells of the *B. cereus* mutants by electroporation (1700 V, Eporator, Eppendorf) to construct the complemented mutants: ∆*gapB*::*gapB*, ∆*gapB*∆*sinR*::*gapB*, ∆*gapB*∆*sinI*::*gapB*, ∆*gapB*∆*sinR*::*sinR*, and ∆*gapB*∆*sinI*::*sinI*. The transformants were cultured on LB plates supplemented with chloramphenicol (20 μg/ml) and incubated at 30°C overnight. The correct complement strain was selected and identified *via* PCR using the primer pairs, 0-9-pMAD-*chi*-s/0-9-pMAD-*chi*-a (Supplementary Table S1). All mutant strains were stored at −80°C and cultivated aerobically in LB broth at 30°C throughout this study.

### Determination of Biofilm Formation

Solid surface-associated biofilm formation was estimated using a crystal violet staining method with some modifications (Zhang et al., 2020a). A single colony of *B. cereus* 0-9 and its transformants was inoculated into 5 ml of LB medium and incubated at 30°C overnight. Three milliliter of LBS medium inoculated by 30 μl of the overnight culture was added into the glass tubes with a diameter of 0.7 cm. And then, the tubes were incubated statically at 30°C for 3 days. The cultures were carefully removed from the tubes letting the pellicle slowly adhere to the tube wall. After gently wash, the pellicle and tube wall twice with deionized water. The remaining cells and matrices in each tube were stained with 3.5 ml of 0.1% (w/v) crystal violet solution for 20 min at 25°C. After washing three times with distilled water, the crystal violet attached to the biofilm was solubilized in 3.5 ml 10% (w/v) sodium dodecyl sulfate. Next, 200 μl of the solution was quantified by measuring the absorbance at 570 nm (Bio Tek, Winooski, VT, United States). The experiment was repeated five times per strain.

### Construction Expression Vectors and Inducible Expression of GapB

The *gapB* gene was amplified using a wild-type *B. cereus* 0-9 template and subcloned into the expression vector, pET28a, before transformation into *E. coli* BL21 (DE3). Recombinant GapB was expressed in *E. coli* BL21 (DE3) strains as previously reported (Zhang et al., 2020a). Briefly, *E. coli* BL21 (DE3) harboring pET28a-*gapB*, growing exponentially in a culture, was induced using 100 μM isopropyl-β-D-thiogalactopyranoside for 4 h at 25°C. Using *E. coli* BL21 (DE3) harboring pET28a vector as control. The cells were harvested by centrifugation and lysed by sonication. After lysis, the crude extracts were centrifuged at 10,000 rpm for at least 30 min. GapB-His6 was purified by nickel-immobilized metal affinity chromatography using a His-Trap column (GE Healthcare). The bound protein molecules were eluted with imidazole before pooling, concentrating, and loading onto a Superdex 200 gel filtration column. The GapB-His6 purity was estimated at >90% *via* SDS-PAGE.

### Determination of GADPH Dehydrogenase Activity

Enzymatic activity assays for GapB were performed as the previous method of Purves et al. (2010) with some modifications. In detail, 5 μg of purified GapB was added to 1 ml of buffer (pH 7.5) containing 50 mM Na_2_HPO_4_, 5 mM EDTA, 20 mM glyceraldehyde-3-phosphate (G-3-P), and either 10 mM NAD^+^ or 2 mM NADP^+^. The sample mixtures were incubated in a 37°C water bath for 30 min. The GapB protein after thermal inactivation was used as the negative control. NAD(P)H production was measured at 340 nm, and an average reading from three wells was recorded for each sample. Each assay was repeated three times on separate days; the results were averaged and are presented alongside the standard error of the data. Values of *p* values were derived using Student’s *t*-test.

### Growth Curve Measurement

The growth curves of the *B. cereus* strains in LBS, KBMS, and M-eps media were determined as follows. A single colony was inoculated into 5 ml of LB medium and grown overnight at 37°C with shaking. Next, 1 ml of overnight culture medium was centrifuged to collect the bacteria, washed three times with sterile water, and then resuspended with different volumes of sterile water to make the OD_600_ value of the tested strains consistent to be 0.6. The suspensions were inoculated into the tested medium at 1:100. The inoculated mediums were grown in the microplates equipped by Bioscreen C (Oy Growth Curves Ab Ltd., Finland) at 37°C statically with oscillating horizontally at 30 min intervals. OD600 value of each micropore was measured every 30 min. The growth curves of the tested strains were recorded automatically.

### Complex Colony Formation Assays

To analyze the colony architecture, we used the method previously reported by Diethmaier et al. (2014) with slight modifications. *Bacillus cereus* strains were precultured in LB medium to an optical density of 0.6–0.8 at 600 nm (OD_600_), and then 1.0 ml of the culture was pelleted and resuspended in 100 μl of sterile supernatant. Approximately 2 μl of this cell suspension was then spotted onto NA plates and incubated at 30°C for 2–3 days. The colonies were photographed using a Digital Single Lens Reflex (Canon EOS 6D, Beijing, China).

### Real-Time Quantitative Reverse Transcription-PCR

Real-time quantitative PCR (qRT-PCR) was performed by using a Cycler instrument (BioRad, Hercules, California, USA) and a Fast Quant RT Kit (Tianjin, China). *Bacillus cereus* 0-9 and its mutants were grown in LBS medium to an OD_600_ of 0.8–1.0 and then harvested. The total RNA was extracted as described previously (Gao et al., 2017). The cDNAs were synthesized using the Fast Quant RT Kitper, according to the manufacturer’s instructions. The GoTaq qPCR Master Mixkit (Tianjin, China) was used for the PCR, according to the manufacturer’s recommended protocol using the primers in Supplementary Table S1. The 16s rRNA was used as endogenous controls and the relative expression of test genes were calculated by the −ΔΔCT value.

### Determination of Extracellular Polysaccharide

After culture for 3 days, 1.0 g biofilms formed by test strains were picked for the determination of EPS content. The biofilms were mixed with 200 μl sterile water and shaken thoroughly to disperse all the aggregates, and then the EPS content in the supernatant of heavy suspension was determined according to previous report (Asadi et al., 2020) with some modifications. For detail, equal volume of 1 M NaOH was added to the supernatant and incubated at 60°C for 1 h. The mixture was centrifuged (10 min and12,000 rpm), and the polysaccharides content in the supernatant was determined by the phenol-sulfuric acid method. Glucose was used as the standard (Masuko et al., 2005). The absorbance was measured using a plate reader at 490 nm (Bio Tek, Winooski, VT, United States), and the averages of the polysaccharide content, measured in triplicate trials, were reported. The supernatant without adding NaOH was used as blank control.

### Determination of Extracellular DNA Content in the Biofilms

Extracellular DNA content in the biofilms was determined using Qubit® dsDNA BR Assay Kits (Thermal Fisher, Shanghai, China). Next, 0.2 g (wet weight) of biofilm from the tested strains was placed into an Eppendorf tube and 200 μl of sterile water was added, and it then oscillated sufficiently to suspend the bacteria cells. The cells were removed by centrifugation at 8,000xg for 5 min. The kits included concentrated assay reagent, dilution buffer, and prediluted DNA standards. The reagent was diluted using the provided buffer, then 20 μl of the liquid supernatant was added and incubated for 2 min, and the concentration of the double-stranded DNA was read using the Qubit® 4 Fluorometer according to the manufacturer’s instructions.

### Transcriptome Analysis

Δ*gapB* and *B. cereus* 0-9 strains were cultured at 30°C, 220 rpm, in LBS medium. When the tested strains grew to the stable period (Culturing for 24 h), the bacterial cells were harvested. The bacteria were quickly frozen in liquid nitrogen, stored in dry ice, and sent to the GENEWIZ Company (Tianjin, China) for chain-specific prokaryotic transcriptome sequencing. The experimental process included RNA extraction, ribosomal RNA removal, library construction and detection, sequencing cluster generation, and on-machine sequencing. Three libraries were prepared by the cDNA from wild-type *B. cereus* 0-9 and Δ*gapB* strain, respectively. Illumina HiSeq sequencing was performed after the qualified libraries were mixed according to the target data volume. Software Bcl2fastq (V2.17.1.14) was used for image base recognition of the original image data of sequencing results. To obtain the original sequencing data (Pass Filter Data), preliminary quality analysis was used as follows. During sequencing, based on the distribution of mass fraction of base position of sample, select analysis results with a quality score value greater than 30 for each Read. For another, Illumina has built-in software based on the mass of the first 25 bases per sequence fragment, then, decide whether the read is retained or discarded. Read count data obtained from gene expression level analysis were used for the input data of gene differential expression. DESeq2 (V1.6.3) of Bioconductor software was used to analyze the genetic differences in this study.

### Live Cell Counts in the Biofilms

Approximately 0.2 g (wet weight) of the biofilms from the tested strains were placed into Eppendorf tubes and suspended in 100 μl of sterile water. After thoroughly blowing and dispersing, the bacteria were collected *via* centrifugation, washed 2–3 times with deionized water, and then suspended with 100 μl deionized water. The number of living cells in the suspension was determined using the viable count method [colony-forming units (CFU)/ml]. The living and dead cells in the suspension were stained with the LIVE/DEAD BacLight Bacterial Viability Kit (Invitrogen, Carlsbad, California, USA) per the manufacturer’s instructions, then 5 μl of stained suspension was dropped on a glass slide, and the ratio of living/dead cells was counted under a fluorescence microscope (Nicon, Tokyo, Japan). Images of the living cells (green fluorescence) and dead cells (red fluorescence) were recorded and combined using the assay software.

### Statistical Evaluations

The vegetative growth curves of the wild-type and mutant strains were generated by plotting the average outcomes (OD_600_) of three experiments per strain. The differences in biofilm formation were analyzed *via* one-way analysis of variance followed by Tukey’s pairwise *post hoc* comparisons.

## Results

### GapB Is Involved in Regulating *Bacillus cereus* 0-9 Biofilm Formation

**Figure 1:**
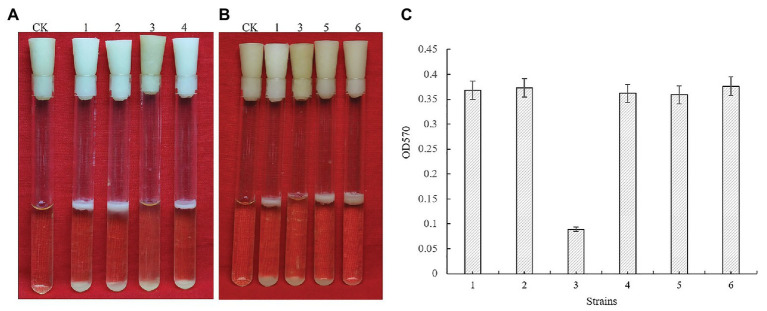
GapB is required for biofilm formation in *Bacillus cereus* 0-9 when it was grown under static conditions in LBS medium. **(A)** The biofilms formed by the three *gap* genes. **(B)** The biofilms formed by *ΔgapB* and its complementary mutants. **(C)** The biofilm production of test strains when they were cultured in LBS medium. Using uninoculated blank medium as control (CK), biofilm formation of the wild-type *B. cereus* 0-9 (1), Δ*gapA* (2), Δ*gapB* (3), Δ*gapN* (4), ∆*gapB/gapB*_pro_ (5), and Δ*gapB*::*gapB*_orf_ (6) strains were imaged after 3 days of growth. Pictures are representative of five experiments.

**Figure 2:**
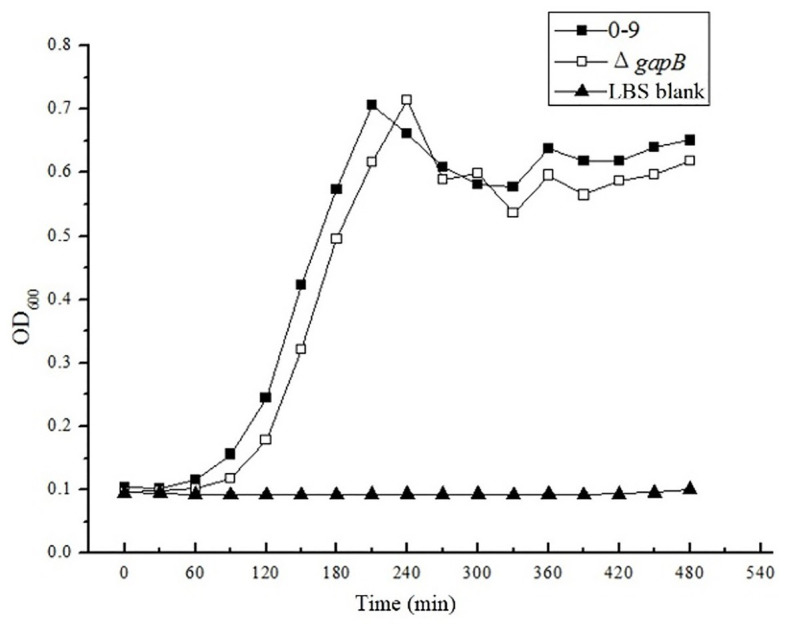
The OD600 value were measured in LBS medium. There is no significantly difference between the growth curve of *Bacillus cereus* 0-9 and Δ*gapB*.

*Bacillus cereus* 0-9 generated biofilm while culturing in the post-stabilization period in both LBS and KBMS media. The overall impression of biofilms formed by *B. cereus* 0-9 and its Δ*gap* mutants were visually record and shown in Figure 1. Biofilm formation of the SL1001 (Δ*gapA*) and SL1003 (Δ*gapN*) mutants could form a normal biofilm structure of *B. cereus* 0-9, but that of SL1002 (Δ*gapB*) mutant was noticeably different in LBS medium. The biofilm formation can take on two forms simultaneously, in the air-liquid (pellicle) and solid-liquid interface, both of which are considered to be crucial biofilms of *B. cereus* 0-9. Δ*gapB* mutant only form a small amount of biofilm at the solid-liquid interface, instead of forming pellicle in the air-liquid interface (Figure 1A). However, the growth curve of the Δ*gapB* mutant did not significantly differ from that of the wild-type (Figure 2). This suggests that the difference in biofilm formation is not due to the difference in growth capacity. The yields of biofilms were quantitatively determined by crystal violet staining, and the results show that Δ*gapB* mutant biofilm yield decreased by 78.5% compared with that of wild-type *B. cereus* 0-9 (Figure 1C). To clarify the relationship between the *gapB* gene and the biofilm phenotype, we constructed two complementary strains of SL7120 (∆*gapB*/*gapB_pro_*) and SL7110 (∆*gapB*::*gapB_orf_*), which supplemented with *gapB via* pAD123-Pgal*-gapB*_pro_ and pMADchi-*gapB*_orf_, respectively, consequently recovering all biofilm yields of the complementary mutants (Figure 1B). Thus, *gapB* may be closely related to *B. cereus* 0-9 biofilm formation.

### Inducible Expression of GapB and Its Enzymatic Activities

**Figure 3:**
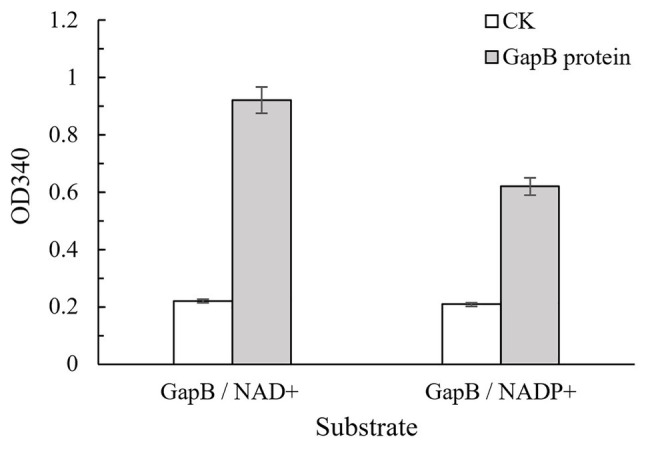
GapB protein has enzyme activities with either NAD^+^ or NADP^+^ as a cofactor. GapB protein after thermal inactivation was used as the negative control (CK). The NAD(P)H production was measured at 340 nm, and an average reading from three wells was recorded for each sample.

GapB can be expressed in *E. coli* BL21 (DE3). The electrophoresis results of the protein expression profile of *E. coli* BL21 harboring the pET28a-*gapB* plasmid was shown in Supplementary Figure S3. GapB weighed 42 kDa, which is the same as the gene annotation. There is no band in the protein expression profile of *E. coli* BL21 harboring the pET28a plasmid, which indicates that the pET28a plasmid has no effect on the GapB expression. GapB was then purified using a cadmium column, and the high-purity protein was used to subsequently determine enzyme activities. Figure 3 shows the Gap protein enzyme activity in the presence of either NAD^+^ or NADP^+^ as cofactors. The *B. cereus* 0-9 Gap proteins had strong dehydrogenase activities, regardless of whether NAD^+^ or NADP^+^ was the cofactor, but the NAD^+^-dependent activity was higher.

### Different Carbon Sources Affect the Growth Curves of *B. cereus* 0-9 and Δ*gapB*

**Figure 4:**
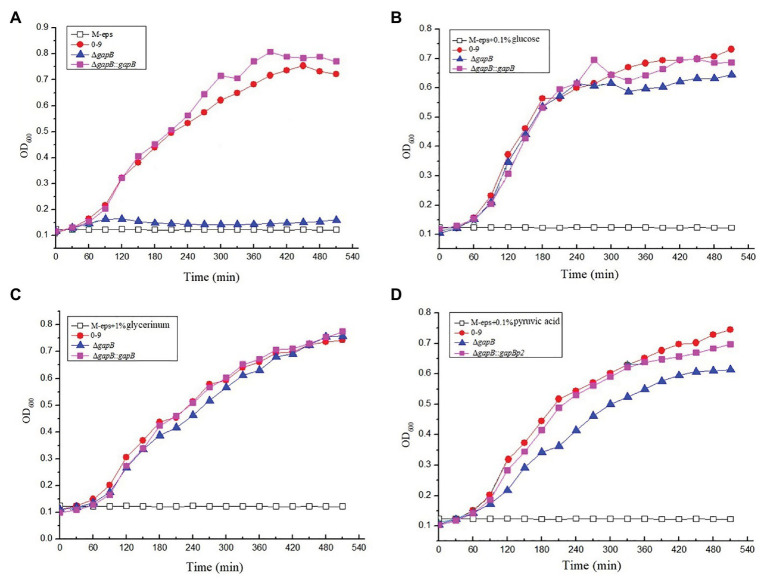
The growth curve of Δ*gapB* and its complementary mutants were measured in M-eps medium with deferent carbon sources of **(A)** M-eps, **(B)** M-eps with 1% glucose, **(C)** M-eps with 1% glycerinum, and **(D)** M-eps with 1% pyruvic acid. The average of three parallel measurements was taken for each reading.

To determine the function of GapB in secondary carbon metabolism, we tested the ability of the Δ*gapB* mutant to use a range of secondary carbon sources. The secondary carbon sources included glucose, the source of glycolysis; pyruvic acid, a key intermediate in the citric acid cycle; and glycerol, which can enter both the glycolytic and gluconeogenic pathways through conversion to glucose-3-phosphatase. Figure 4 shows the growth curve of Δ*gapB* and its complementary mutants in M-eps medium with different carbon sources. Our results revealed where various carbon sources enter either glycolysis or gluconeogenesis. Wild-type *B. cereus* 0-9 grows well in M-eps medium, but Δ*gapB* does not. When the *gapB* gene was complemented, the growth curve of the Δ*gapB*::*gapB* mutants completely recovered to that of wild strain (Figure 4A). Adding glucose acid to the M-eps medium also recovered the Δ*gapB* growth curve, only with slightly weaker in the stable period (Figure 4B). Adding glycerol to the medium completely recovered the growth ability of the Δ*gapB* strain (Figure 4C). Supplementing pyruvic acid in the M-eps medium partly recovered the Δ*gapB* proliferation, and the growth ability was significantly lower than that of wild-type *B. cereus* 0-9 (Figure 4D). Thus, we concluded that the role of GapB in the glycolysis pathway may play a key role in gluconeogenesis.

### Expression Level of *pgm* in the Δ*gapB* Mutant Determined *via* qRT-PCR

The *pgm* gene encodes allosteric glucose-6-phosphatase, which catalyzes glucose-6-phosphate to glucose-1-phosphate, an irreversible catalytic reaction in the gluconeogenic pathway (Goto et al., 2016). To confirm the function of GapB in *B. cereus* 0-9 gluconeogenesis, we measured the transcription level of *pgm*, which is related to the allostery of phosphorylated glucose in gluconeogenesis, *via* qRT-PCR. When cultured for 6 h in LBS medium, the transcription level of *pgm* in the Δ*gapB* mutant did not significantly differ (*p* > 0.5) from that of wild-type *B. cereus* 0-9. However, incubating Δ*gapB* to a stable level for 12 h, downregulated its *pgm* transcription by 32-fold, indicating that deleting g*apB* blocked the gluconeogenic pathway in *B. cereus* 0-9 and inhibited the Pgm protein activity, thus affecting glucose-1-phosphate synthesis. These results demonstrated that the GapB is a key factor controlling gluconeogenesis in *B. cereus* 0-9.

### Different Carbon Sources Affect the Δ*gapB* Biofilm Yield

**Figure 5:**
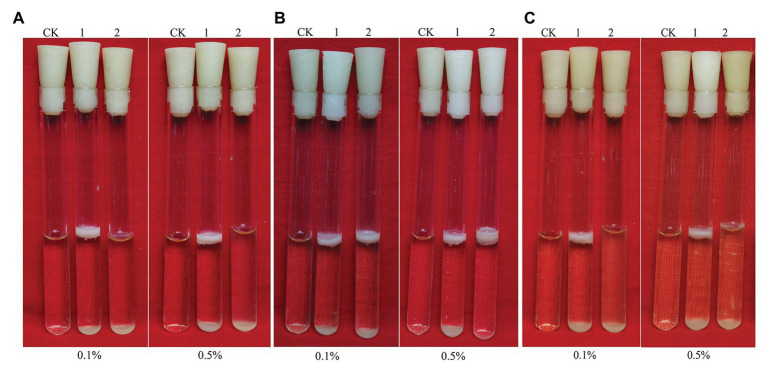
Biofilms of *Bacillus cereus* 0-9 (1) and Δ*gapB* (2) formed in LBS medium with different carbon sources of **(A)** glucose, **(B)** glycerinum, and **(C)** pyruvic acid, using uninoculated blank medium as control (CK). Biofilms were imaged after 3 days of growth. Pictures are representative of five experiments.

Figure 5 shows the biofilm formation of Δ*gapB* and its complementary mutants in LBS medium with different carbon sources. Adding glucose or pyruvate to LBS did not recover the Δ*gapB* biofilm formation (Figures 5A,C), but the growth curve of Δ*gapB* in LBS did not significantly differ from *B. cereus* 0-9 (*p* < 0.05; Figure 2). Glycerinum supplementation recovered the Δ*gapB* biofilm formation to the same level as that of wild-type *B. cereus* 0-9 for concentrations ranging from 0.1–0.5% (Figure 5B). Thus, we concluded that the *B. cereus* 0-9 biofilm formation requires sufficient glucose-1-phosphatase, and *gapB* deletion blocking gluconeogenesis, which leads to abnormal biofilm formation. Supplementing glycerol in the medium enabled gluconeogenesis *via* the glycerol branch, which circumvented the limitation of the *gapB* deletion. This is consistent with the findings for *S. aureus* reported by Purves et al. (2010).

### Effects of *glk* and *gapB* Gene Deletion on *B. cereus* 0-9 Biofilm Formation

**Figure 6:**
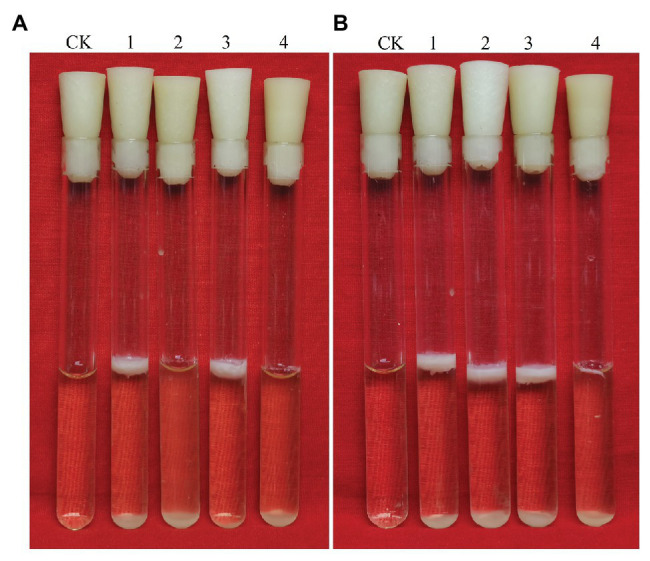
Biofilms of *Bacillus cereus* 0-9 (1), Δ*gapB* (2), Δ*glk* (3), and Δ*glk*Δ*gapB* (4) mutants in LBS medium with different carbon sources of **(A)** glucose and **(B)** glycerinum were imaged after 3 days of growth, using uninoculated blank medium as control (CK). Pictures are representative of five experiments.

**Figure 7:**
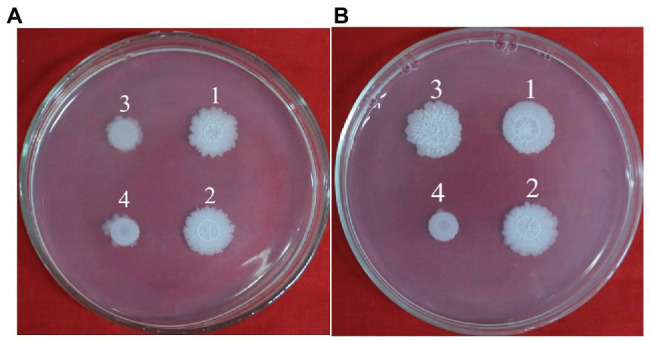
Colony morphology of *B. cereus* 0-9 (1), Δ*glk* (2), Δ*gapB* (3) and Δ*glk*Δ*gapB* (4) strains were determined in NA medium with 1% glycerinum **(B)**, using NA medium without glycerinum as control **(A)**. Pictures are representative of three experiments.

The *glk* gene encodes glycerol kinase, which catalyzes the phosphorylation of glycerin to glycerin-3-phosphate, which is then dehydrogenated to dihydroxyacetone phosphate and converted to glyceraldehyde triphosphate to participate in glycometabolism or gluconeogenesis (Park et al., 2000). Thus, deleting *glk* would block the glycerol branch in the gluconeogenic pathway. To determine the function of GapB in *B. cereus* 0-9, we studied the biofilm formation and colony morphology of SL1004 (Δ*glk*), Δ*gapB*, SL2003 (Δ*glk*Δ*gapB*), and wild-type *B. cereus* 0-9. Our experimental results show that only *glk* gene deletion alone would not affect the *B. cereus* 0-9 biofilm formation. When *gapB* gene was deleted, the biofilm formation of *B. cereus* 0-9 was repressed. Supplementing glycerinum in the medium completely recovered the biofilm formation of Δ*gapB*, but the double gene mutant, Δ*glk*Δ*gapB*, still could not restore the biofilm formation abilities (Figure 6). When the gluconeogenesis was blocked by deletion of *gapB* gene, glycerol was transferred into the gluconeogenic pathway *via* dihydroxyacetone phosphate to supplement the function of GapB. However, the *glk* gene deletion can block glycerin into the glycometabolism, so the double knockout strain of Δ*glk*Δ*gapB* cannot restore the gluconeogenesis, even in the presence of glycerol. Therefore, these results indicated that the gluconeogenesis plays an important role in the regulation of the biofilm formation of *B. cereus* 0-9, and the GapB is a key enzyme in the control of glucogenesis, so it is crucial for the formation of biofilms. Further, we determined the colony morphology on solid NA plates with or without 1% glycerinum (Figure 7). The Δ*glk* colony morphology did not noticeably differ from the that of wild-type *B. cereus* 0-9, regardless of whether the plate contained 1% glycerinum. After knocking out *gapB*, the colonies became smaller, their folds became less uniform, and most of the surrounding areas became smooth and flat (Figure 7A). Supplementing 1% glycerinum in the plates restored the Δ*gapB* colony morphology, but the *glk* and *gapB* double mutants did not recover the colony morphology on the same plates (Figure 7B). This is consistent with the results for the biofilm formed by these tested strains in liquid LBS medium. Hence, we considered that the GapB is also critical to *B. cereus* 0-9 colony morphology and may be an important factor in biofilm formation on the surfaces of solid substrates. Therefore, we concluded that the GapB influenced *B. cereus* 0-9 biofilm formation by regulating gluconeogenesis.

### The SinI/R System Is Involved in *Bacillus cereus* 0-9 Biofilm Formation

**Figure 8:**
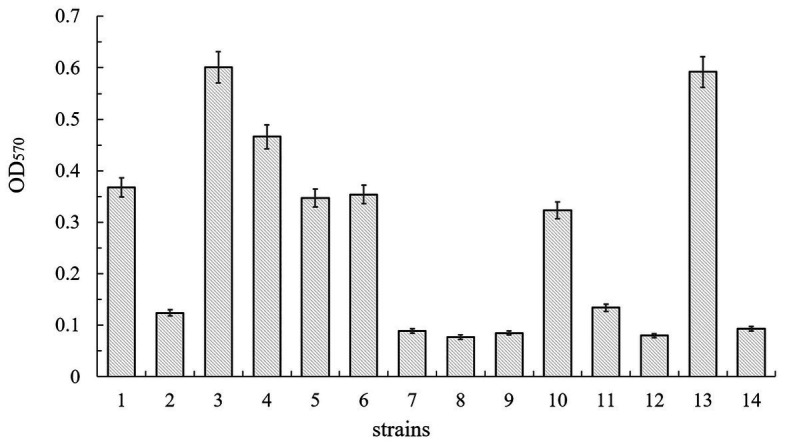
Biofilm production of *B. cereus* 0-9 (1), Δ*sinI* (2), Δ*sinR* (3), Δ*sinI*Δ*sinR* (4), Δ*sinI*::*sinI* (5), Δ*sinR*::*sinR* (6), Δ*gapB* (7), Δ*gapB*Δ*sinI* (8), Δ*gapB*Δ*sinR* (9), Δ*gapB*::*gapB* (10), Δ*gapB*Δ*sinI*::*gapB* (11), Δ*gapB*Δ*sinI*::*sinI* (12), Δ*gapB*Δ*sinR*::*gapB* (13), and Δ*gapB*Δ*sinR*::*sinR* (14) were measured by crystal violet staining. The average of three parallel measurements was taken for each reading.

To determine whether the SinI/R system is involved in *B. cereus* 0-9 biofilm formation, we constructed FPU1051I (Δ*sinI*) and SL1052R (Δ*sinR*) mutants and measured their biofilm yields (Figure 8). The biofilm formation ability of Δ*sinI* was decreased by 66.30% compared with that of wild-type *B. cereus* 0-9. Conversely, the biofilm formation ability of the Δ*sinR* mutants was increased by 63.32%; therefore, the SinI/R system also exists in *B. cereus* 0-9. The repressor SinR is central to the regulatory switch in biofilm formation in *B. subtilis* (Diethmaier et al., 2014), whose function and mechanism in regulating biofilm formation has been clearly revealed (Chu et al., 2006; Newman et al., 2013). To examine the mechanism of the SinI/R system in regulating *B. cereus* 0-9 biofilm formation, we measured the transcription levels of several biofilm formation-related genes *via* qRT-PCR. Culturing Δ*sinR* for 4 h upregulated the transcription levels of *tasA*, *calY*, and *sipW* by 147.03-, 630.35-, and 445.72-fold, respectively, compared with that of wild-type *B. cereus* 0-9 (Supplementary Table S2). These results indicated that the mechanism by which the SinI/R system regulates biofilm formation in *B. cereus* 0-9 may the same as that of *B. subtilis*.

### GapB Do Not Affect Biofilm Formation Through the SinI/R System

To determine whether GapB-mediated inhibition of biofilm formation depends on the presence or absence of SinI/R, the biofilm yield was measured using the Δ*gapB*, SL2001 (Δ*gapB*Δ*sinI*), and SL2002 (Δ*gapB*Δ*sinR*) gene mutants and their complementary strains. Figure 8 shows the relative biofilm formation ratios by the experimental strains. Compared with that of *B. cereus* 0-9, the biofilm formation ability of the Δ*gapB* mutant was decreased by 75.82%. The biofilm yield of the double-knockout strains, Δ*gapB*Δ*sinI* and Δ*gapB*Δ*sinR*, were decreased by 79.08 and 76.90%, respectively. Among the complementary mutants, the biofilm yields of SL7251I (Δ*gapB*Δ*sinI*::*sinI*) and SL7252R (Δ*gapB*Δ*sinR*::*sinR*) were also decreased by 78.26 and 74.73%, respectively. Conversely, the biofilm yield of SL7212 (Δ*gapB*Δ*sinR*::*gapB*) was increased by 60.87%, which was the same as that of Δ*sinR*, and the biofilm yield of SL7211 (Δ*gapB*Δ*sinI*::*gapB*) was decreased by 63.59%, which was the same as that of Δ*sinI*. Thus, we concluded that once *gapB* is lost, wild-type *B. cereus* 0-9 can no longer form biofilm normally, regardless of whether *sinI* and *sinR* are present. In other words, *gapB* deletion does not affect the SinI/R system function in *B. cereus* 0-9. And it is possible that the absence of *gapB* is dominant over the absence of *sinR* under this experimental condition.

GapB expression in mutant strains without *sinR* was detected by western blotting (Supplementary Figure S4). GapB was not expressed in the Δ*gapB* or Δ*gapB*Δ*sinR* mutants but was expressed normally in the Δ*sinR* mutant, which is similar to that of wild-type *B. cereus* 0-9, indicating that the *sinR* does not affect GapB expression.

### EPS and Extracellular DNA Content in Biofilms

The polysaccharides content in the biofilms were determined by the phenol-sulfuric acid method. According to our experiment results, the EPS contents in the biofilms of *B. cereus* 0-9 and Δ*gapB* mutant were 45.64 and 46.10 mg/g (WW), respectively (Supplementary Table S3). This suggests that the *gapB* gene deletion will not cause changes in the production of exopolysaccharides in *B. cereus* 0-9 biofilm. Therefore, it is very possible that the biofilm defect of Δ*gapB* was irrelated to the production of exopolysaccharides.

Table 2 shows the detection results for extracellular DNA in the biofilms. The extracellular DNA content in the biofilms formed by Δ*gapB* was 0.099 μg/mg, while that of wild-type *B. cereus* 0-9 reached 1.677 μg/mg. Thus, *gapB* deletion reduced the amount of extracellular DNA released. Additionally, the DNA contents of the biofilms formed by Δ*gapB*/*gapB_pro_* and Δ*gapB*::*gapB_orf_*, in which *gapB* gene was complemented, were recovered to the levels of wild-type *B. cereus* 0-9. This indicated that the change in biofilm yield due to *gapB* deletion was positively correlated with the extracellular DNA content in the biofilms. Vilain et al. (2009) reported that the *B. cereus* requires extracellular DNA to form biofilms. Therefore, we concluded that the GapB likely regulates biofilm formation of *B. cereus* 0-9 by controlling the release of extracellular DNA.

**Table 2:** Extracellular DNA content in the biofilms.

Strains	Wet-weight of biofilm (g)	eDNA content of tested sample (μg/ml)	eDNA content of biofilm (μg/g)
*B. cereus* 0-9	0.26	43.60 ± 0.517	167.7
Δ*gapB*	0.27	2.66 ± 0.462	9.9
Δ*gapB*::*gapB_orf_*	0.22	37.10 ± 0.331	168.6
Δ*gapB*/*gapB_pro_*	0.27	46.60 ± 0.163	172.6

“eDNA” represents extracellular DNA.

### Deletion of *gapB* Induces Upregulation of the Autolysis Regulator LrgAB in the Stable Phase

Cell grown to stationary phase are the source for biofilm formation in the solid-liquid interface in static conditions. We hypothesized that the differences in the ability to form biofilms between the wild-type *B. cereus* 0-9 and its Δ*gapB* mutants, stem from changes in their transcriptomic profile at the stationary growth phase. Our results showed that only 18 genes differed significantly after knocking out *gapB*, among which, six genes were upregulated, and 12 genes were downregulated (Table 3). The gene with the most significantly different expression level was the operon *lrgAB* (containing *lrgA* and *lrgB*). LrgAB was previously reported to be a regulatory system closely related to autolysis of *S. aureus* NCTC8325 (Chu et al., 2013), and it negatively regulates extracellular murein hydrolase activity; thus, it can inhibit the autolysis of *S. aureus* (Groicher et al., 2000). LrgAB likely plays a similar role in *B. cereus* 0-9, which explains why less extracellular DNA exists in the Δ*gapB* mutant biofilm than in that of wild-type *B. cereus* 0-9.

**Table 3:** Genes with significantly different expression levels in Δ*gapB* mutants.

Gene name	Log (2^Fold Change^)	Annotation
FRY47_26740	2.62	Antiholin-like protein LrgA
FRY47_26735	2.07	Antiholin-like protein LrgB
FRY47_02510	−1.98	PTS Acetylglucosamine transporter subunit IIB
FRY47_11490	1.95	MFS transporter
FRY47_26815	−1.48	GMP reductase
FRY47_21465	−1.30	DoxX family protein
FRY47_25220	−1.52	Aldehyde dehydrogenase
FRY47_05025	−1.49	Dihydroxyacetone kinase transcriptional activator DhaS
FRY47_23430	−1.46	PepSY domain-containing protein
FRY47_22570	−1.38	S-Adenosylmethionine decarboxylase proenzyme
FRY47_09710	1.29	Hypothetical protein
FRY47_11235	1.27	YokU family protein
FRY47_12105	−1.12	Hypothetical protein
FRY47_02910	−1.26	Hypothetical protein
FRY47_00975	1.16	Hypothetical protein
FRY47_04370	−1.07	DMT family transporter
FRY47_20510	−1.06	3-Methyl-2-oxobutanoate dehydrogenase subunit alpha
FRY47_20500	−1.06	2-Oxo acid dehydrogenase subunit E2

### Dead and Living Cells in Biofilms

**Figure 9:**
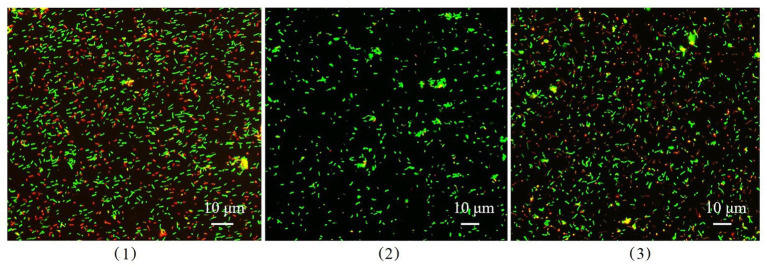
The image of dead and living bacterial cells in biofilms of *Bacillus cereus* 0-9 (1), Δ*gapB* (2), and Δ*gapB*::*gapB* (3) observed by fluorescence microscopy. Green fluorescence indicates the live bacterial cells and red ones are the dead cells.

Dead and living bacterial cells in biofilms were observed using a fluorescence microimaging system (Figure 9). Dead cells (red) in the biofilm of wild-type *B. cereus* 0-9 constituted nearly half the total cells in the suspension, but the Δ*gapB* mutant contained only a few dead cells. To confirm the difference between the dead and living cells in the biofilm, we determined the number of living bacteria per unit wet weight of the biofilms by the viable count method, and the results were shown in Table 4. The “colony number” in Table 4 means 100 μl of bacterial suspension of biofilm cells was diluted to 10^−8^, then, 100 μl of this diluent was coated on a counting plate, and the number of bacterial colonies was calculated after culturing for 24 h. The viable count was 24.65 × 10^9^ CFU per gram (wet weight) in the *B. cereus* 0-9 biofilms. However, the viable count reached 181.88 × 10^9^ CFU per gram in the Δ*gapB* biofilms, which was nearly 7.5 times than that of *B. cereus* 0-9. This result is consistent with that of the transcriptome analysis, in which *lrgAB* expression was the most upregulated in the Δ*gapB* mutant. Therefore, it is very possible that the GapB was involved in extracellular DNA release and biofilm formation by regulating the expression of LrgAB.

**Table 4:** Counts of living bacteria in the biofilms.

Strains	Wet-weight of biofilms (g)	Suspension volume (μl)	Colony number (×10^8^ CFU)	Results (×10^9^ CFU/g)
*B. cereus* 0-9	0.20	100	49.3	24.65
Δ*gapB*	0.16	100	291	181.88
Δ*gapB*::*gapB*	0.24	100	63.3	26.375

### LrgAB Is Involved in the Biofilm Formation of *Bacillus cereus* 0-9

**Figure 10:**
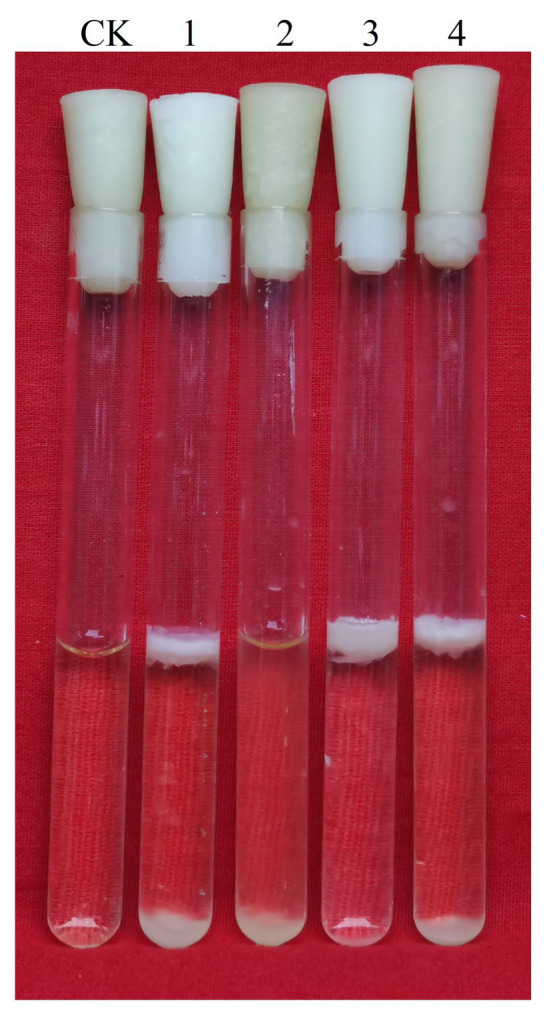
Biofilms of *Bacillus cereus* 0-9 (1), Δ*gapB* (2), Δ*lrgAB* (3), and Δ*gapB*Δ*lrgAB* (4) mutants in LBS medium were imaged after 3 days of growth, using uninoculated blank medium as control (CK). Pictures are representative of five experiments.

We simultaneously knocked out *lrgA* and *lrgB* genes to construct Δ*lrgAB* and Δ*gapB*Δ*lrgAB* mutant strains, and the determination results of their biofilm formation states are shown in Figure 10. It shows that the biofilm production of Δ*lrgAB* is higher than that of wild strains in the LBS medium. The biofilm formation ability of Δ*gapB*Δ*lrgAB* mutant recovered, but its biofilm production is lower than that of Δ*lrgAB* mutant. This suggests that the LrgAB is indeed related to the biofilm formation of *B. cereus* 0-9, and the GapB is very possible to be the upstream regulator of LrgAB. Due to lack of regulatory proteins that maintain the vitality of bacterial cells, Δ*lrgAB* mutant strain may release a large number of DNA. Thus, a huge accumulation of eDNA making the biofilm production increases. These results may further support our view of “GapB is involved in extracellular DNA release and biofilm formation dependent on regulating the expression or activities of LrgAB.”

## Discussion

### Deleting *gapB* Affected *B. cereus* 0-9 Biofilm Formation

*Bacillus cereus* 0-9 is a highly adaptable bacterium that is widely distributed in soil, water, plants, and wheat rhizospheres (Kang et al., 2019). Our previous study showed that the wild-type *B. cereus* 0-9 isolates can form pellicles on air-liquid interfaces and architecturally complex colonies on solid surfaces (Zhang et al., 2020a). This constructive survival strategy enables bacteria to use a greater variety of nutrients, endure rapid environmental changes, and resist multiple adverse threats (Zhang et al., 2020b). Previous reports showed that the GapA, a glyceraldehyde-3-phosphate dehydrogenase that plays a key role in glycometabolism pathways, is involved in the biofilm formation of *S. aureus* and *B. subtilis* (Purves et al., 2010; Romero et al., 2014). In our previous research, we often found that in solid medium with different carbon sources, the bacterial colony folds formed by the same bacteria are significantly different, which is also a form of biofilm existence. It is speculated that some enzymes of glucometabolism pathway may be involved in the formation of bacterial biofilm. GAPDH, as a kind of moonlighting protein, not only plays a role in the process of glucose metabolism, but also has a certain regulatory effect on the pathogenic capacity of bacteria (Giménez et al., 2014). More recent research shows that the glucose effect on *Candida albicans* biofilm during tissue invasion (Figueira et al., 2020). Therefore, in this study, we aimed to investigate whether GAPDH, a key regulator of glucose metabolism, affects biofilm formation in *B. cereus* 0-9. Whole-genome sequencing (GenBank: CP042874.1) and subsequent gene function annotation revealed that three genes encode GAPDH in the *B. cereus* 0-9 genome: *gapA*, *gapB*, and *gapN*. Thus, we knocked out the *gap* genes and measured the biofilm yields of these gene deletion mutants. We do not know which *gap* gene may be related to the regulation of biofilm formation. Therefore, mutant strains of each *gap* gene were constructed separately, and the production of each biofilm was measured under the same conditions. Our results showed that the biofilm yields of the Δ*gapB* mutant were decreased by 78.5%, but deletion of *gapA* and *gapN* did not affect *B. cereus* 0-9 biofilm formation. Thus, we further studied the function of *gapB* to illustrate the mechanism by which *gapB* regulates *B. cereus* 0-9 biofilm formation.

### GapB Is a Glyceraldehyde-3-Phosphate Dehydrogenase and a Key Regulator of Gluconeogenesis

We cloned the *gapB* gene and expressed GapB protein in *E. coli* BL21 (DE3). Measuring the dehydrogenase activity of purified GapB showed that the GapB is a glyceraldehyde-3-phosphate dehydrogenase with strong dehydrogenase activities, regardless of whether NAD^+^ or NADP^+^ is a cofactor. *Bacillus cereus* possesses two GAPDH activities, which are NAD^+^- and NADP^+^-dependent, catalyzed by two enzymes with distinct coenzyme specificities and different phosphate regulation patterns (Abdelghani et al., 2002). Our results indicated that the GapB in *B. cereus* 0-9 has both NAD^+^- and NADP^+^-dependent dehydrogenase activity. Purves et al. (2010) previously reported that the GapB in *S. aureus* lacks NAD^+^-dependent dehydrogenase activity. Thus, although GapB plays an important role in glucose metabolism in different bacterial strains, some functions vary among strains. Giménez et al. (2014) reported that GapB, as a GAPDH, is a moonlighting protein in bacteria, which has unrelated and independent functions.

The Gap protein, a GAPDH, catalyzes the conversion between glyceraldehyde-3-phosphate and 1,3-diphosphate glycerate and is a key enzyme in the glycolytic pathway (Abdelghani et al., 2002). Our results showed that the *B. cereus* 0-9 grew well in M-eps medium, but Δ*gapB* could not, unless 1% glucose or glycerol was added. This suggests that the GapB may play an important role in gluconeogenesis, and the loss of anabolic carbon metabolism is most likely due to disruption of the gluconeogenesis pathway. Interestingly, the Δ*gapB* mutant could grow with glycerol as the primary carbon source, indicating that the GapB must play key roles before this stage in the gluconeogenic pathway for glycerol to recover the growth defect of the Δ*gapB* mutant. To confirm the function of *gapB*, we further measured the transcription level of *pgm* gene, which is related to the allostery of phosphorylated glucose in gluconeogenesis (Goto et al., 2016). Consequently, *pgm* expression was downregulated 32-fold when *gapB* was deleted, indicating that deleting *gapB* blocked the gluconeogenic pathway of *B. cereus* 0-9, thus inhibiting glucose-1-phosphate synthesis, thereby affecting the synthesis of polysaccharides and other bacterial phenotypes.

### GapB Is Closely Related to *B. cereus* 0-9 Biofilm Formation

*Bacillus cereus* displays highly diverse lifestyles and ecological niches. It can produce various biofilms that differ in their architecture and formation mechanisms, possibly reflecting an adaptation to various environments (Majed et al., 2016). We determined the biofilm yield of Δ*gapB* strain and discovered that their biofilm formation ability was dramatically decreased. However, when the *gapB* gene was complemented, the biofilm formation ability of Δ*gapB*::*gapB* mutants could be recovered. Thus, in addition to being a GAPDH, GapB has some environmental stress resistance functions such as biofilm formation.

Interestingly, supplementing the medium with 0.1–0.5% glycerol restored the biofilm formation capacity of the Δ*gapB* mutants, but glucose and pyruvate did not (Figure 5). Carbon source decomposable metabolite repression (activation) is a conserved regulatory pathway of carbon source metabolism in bacteria, which ensures the orderly utilization of different carbon sources by bacteria and improves the growth rate and environmental adaptability of bacteria (Lorca et al., 2005). In G^+^ bacteria, including *Bacillus*, carbon catabolite repression (CCR) functions mainly through carbon decomposition metabolite repressor protein CcpA. In the presence of a preferred carbon source of glucose, fructose-1,6-diphosphate (FBP), as a product of glycolysis intermediate, can stimulate the bifunctional enzyme HprK/P to perform kinase activity, causing the phosphorylation of Hpr protein to produce Hpr (ser46 ~ P). Hpr binds to CcpA to form complexes that bind to CRE-sites and repress or activate transcription of metabolic genes from other carbon sources. After glucose depletion, HprK/P exerts phosphatase activity, resulting in dephosphorylation of Hpr (ser46 ~ P), inability to bind to CcpA to form complexes, and loss of regulatory function (Voort et al., 2008; Tomoyasu et al., 2010). In addition, the other two CcpA-independent carbon repressor proteins, CcpN and CcpC, inhibit gluconeogenesis and TCA cycles in the presence of glucose, ensuring the priority of glycolysis (Voort et al., 2008). Due to the conservative regulatory mechanism found above, glycolysis takes precedence after glucose addition, so the production of biofilms by Δ*gapB* mutants cannot be restored. The related glycerol metabolism genes *glpF* (glycerol uptake facilitator) and *glpK* (glycerol kinase) are strictly regulated by CcpA and can be expressed only when glucose utilization is completed. Therefore, when glucose is exhausted and glycerol is present, glycerol enters the cell under the action of glycerol kinase and glycerol triphosphate dehydrogenase, and is converted into dihydroxyacetone phosphate and glycerol triphosphate simultaneously, entering the glycolysis and gluconeogenesis pathways, restoring the biofilm defect of Δ*gapB*.

The *glk* gene encodes glycerol kinase, which catalyzes the phosphorylation of glycerin to glycerin-3-phosphate. Colony morphology assays of Δ*glk*, Δ*gapB*, and Δ*glk*Δ*gapB* revealed that the Δ*glk* colony morphology did not noticeably differ from that of wild-type *B. cereus* 0-9. When *gapB* was knocked out, the colonies became smaller and the colony folds became less uniform (Figure 7A). Adding 1% glycerinum to the plates restored the Δ*gapB* colony morphology, but did not restore the double mutant, Δ*glk*Δ*gapB*, which is on the same plates (Figure 7B). It indicated that the GapB is a key factor of controlling gluconeogenesis in *B. cereus* 0-9, and glycerol supplementation in the culture medium can remove the restriction induced by deleting *gapB*. Therefore, we concluded that the *gapB* is closely related to *B. cereus* 0-9 biofilm formation and is likely linked by GapB-regulated gluconeogenesis. However, how GapB is involved in regulating biofilm formation is unclear. Exopolysaccharides are important substrates for *B. cereus* biofilm formation (Majed et al., 2016). GapB, as a key regulator of gluconeogenesis, may regulate glucose-1-phosphate synthesis when carbon sources are scarce, thereby affecting the synthesis of polysaccharides and other bacterial phenotypes. Surprisingly, however, *gapB* gene deletion caused no change on EPS production of *B. cereus* 0-9 in LBS medium (Supplementary Table S3). Therefore, the mechanism by which GapB regulates biofilm formation should be further explored.

### GapB Did Not Affect Biofilm Formation Through the SinI/R System

Biofilm formation requires a complex regulatory pathway that coordinates gene expression with external environmental conditions and structural components involved in assembling the protective extracellular matrix (Romero, 2013; Vlamakis et al., 2013). The SinI/R system is the most clearly reported system for regulating *B. cereus* biofilm formation (Newman et al., 2013). SinR is a DNA-binding protein that represses the transcription of operons involved in producing both exopolysaccharides and the protein component of the matrix (Branda et al., 2001; Diethmaier et al., 2014). In *B. subtilis* and *B. cereus*, three proteins, TapA, TasA, and SipW, are required for biofilm formation (Caro-Astorga et al., 2015; Candela et al., 2019). SinR represses transcription of the *tapA-sipW-tasA* operon (Kearns et al., 2005). Gene annotations show that both *sinI* and *sinR* exist in the *B. cereus* 0-9 genome. Our results showed that the biofilm formation ability of Δ*sinI* was decreased by 66.30%, and that of the Δ*sinR* mutants was increased by 63.32% compared with that of wild-type *B. cereus* 0-9. Knocking out *sinR* upregulated *tasA*, *calY*, and *sipW* transcription levels by 147.03-, 630.35-, and 445.72-fold, respectively (Supplementary Table S2). Therefore, the SinI/R system exists in *B. cereus* 0-9 and regulates its biofilm formation as previously reported by Newman et al. (2013).

We further constructed a series of double-knockout strains and their complementary mutants and measured their biofilm yields. The mutant strains without *gapB* showed a similar ability to form biofilms as that of Δ*gapB*, regardless of whether *sinR* and *sinI* were present (Figure 8). When *gapB* was complemented, Δ*gapB*Δ*sinI*::*gapB* showed the same ability to form biofilms as did Δ*sinI* and as did Δ*gapB*Δ*sinR*::*gapB* with Δ*sinR*. This indicates that the GapB is not involved in regulating the SinI/R system. Furthermore, GapB was expressed in the Δ*sinR* mutant. Therefore, we concluded that GapB may affect biofilm formation through another regulatory mechanism but not through the SinI/R system.

### GapB Affects Biofilm Formation by Regulating Extracellular DNA Production

Bacteria are usually embedded in a self-produced matrix to form biofilm, and the complex architecture of a biofilm is maintained by an extracellular matrix of EPSs, proteins, and eDNA (Flemming and Wingender, 2010; Candela et al., 2019). The function of eDNA in biofilms has attracted much attention in recent years (Okshevsky et al., 2015; Saunders et al., 2020). *Bacillus cereus* follows this rule, and its matrix contains all three components (Majed et al., 2016). GapB regulates *B. cereus* 0-9 biofilm formation independent of EPSs and regulatory proteins in the SinI/R system, which is likely related to the release of DNA in the matrix. Exponential-phase cells of *B. cereus* are reportedly decorated with extracellular DNA, and biofilm formation requires DNA as part of the extracellular polymeric matrix (Vilain et al., 2009). *Bacillus cereus* can form DNA-containing biofilms on glass surfaces exposed to exponentially growing bacterial populations. Thus, we measured the extracellular DNA content in the biofilms formed by *B. cereus* 0-9 and its Δ*gapB* mutant and discovered that deleting *gapB* decreased the DNA content in the biofilms from 167.7 to 9.9 mg/g, indicating that the change in biofilm yield caused by deleting *gapB* was positively correlated with the extracellular DNA content in the biofilms. Therefore, GapB is involved in regulating biofilm formation, likely by controlling the release of extracellular DNA. However, whether this extracellular DNA comes from cell lysis remains uncertain.

### GapB Participated in Regulating Biofilm Formation Through LrgAB

To explore the mechanism by which GapB is involved in regulating extracellular DNA production in *B. cereus* 0-9 biofilm, we sequenced the transcriptomes of the Δ*gapB* mutants. The gene that differed the most significantly between *B. cereus* 0-9 and Δ*gapB* was the *lrgAB* gene, which is annotated as an operon. LrgAB was previously reported to play a key role in autolysis, biofilm formation, glucosyltransferase expression, and oxidative stress tolerance (Ahn et al., 2010). LrgAB negatively regulates extracellular murein hydrolase activity and is a type of regulatory system closely related to the autolysis of *S. aureus* (Chu et al., 2013). To prove whether the biofilm formation defect of Δ*gapB* is related to the lysis of bacterial cells in biofilm, we counted the viable bacteria in per unit of wet-weight biofilms and found that the number of living cells in the biofilm formed by Δ*gapB* mutant was nearly 7.5 times more than that of wild-type *B. cereus* 0-9 (Table 4). Observing the ratio of dead to living cells in the biofilm using a fluorescence microimaging system confirmed this result (Figure 9). Thus, we concluded that the biofilm formation defect of Δ*gapB* is related to bacterial cell autophagy and lysis, which is regulated by LrgAB. This conclusion is inconsistent with that of Vilain et al. (2006), who considered the *B. cereus* biofilm extracellular DNA did not originate primarily through cell lysis; this conclusion was speculative and not directly evidenced. We further knocked out *lrgAB* gene, and found that the Δ*lrgAB* can form more biofilms than wild-type *B. cereus* 0-9 at the same conditions. It means that the deletion of *lrgAB* inevitably induces bacterial cell autophagy and lysis, so, it leads to more extracellular DNA existing in the biofilms of Δ*lrgAB* than in that of *B. cereus* 0-9. The biofilm production of Δ*gapB*Δ*lrgAB* was recovered to that of *B. cereus* 0-9, but Δ*gapB* (Figure 10). Therefore, we concluded that deleting *gapB* would likely induce the expression of *lrgAB* to prevent autolysis of the Δ*gapB* cells, thus reducing the release of extracellular DNA and affecting its biofilm formation. It also indicated that the GapB is located in the upstream of *lrgAB*, and it is involved in extracellular DNA release and biofilm formation of *B. cereus* 0-9 by regulating the expression or activities of LrgAB. However, the biofilm production of Δ*gapB*Δ*lrgAB* is lower than that of Δ*lrgAB* mutant. It means that the absence of GapB also affects biofilm formation through any mechanisms different from regulation of *lrgAB*. Transcriptional sequencing results show that in addition to *lrgA* and *lrgB*, there are significant differences in the expression levels of other 10 genes, which is also indicating that the GapB is involved in the regulation of various gene expressions, not only *lrgAB*. At present, there are still many interesting mysteries about GapB, which will attract us to explore it further in our future study.

In addition to its glycolytic activity, GAPDH is implicated in regulation of proliferation, autophagy, maintenance of highly tumorogenic cancer cells, development of apoptosis, and many other processes (Lazarev et al., 2016). However, how GAPDH is linked to bacterial autolysis or modified cell death is unclear. When GAPDH molecules are inactivated by oxidation or S-nitrosylation, they may bind polypeptides, such as Siah1 ubiquitin ligase, and transport them to the nucleus (Hara et al., 2006). Other studies have reported that an extracellular *Streptococcus* GAPDH can interact with C5, a component of the complement system, which promotes its degradation, in coordination with the bacterial surface protease (Giménez et al., 2014). This strategy allows the pathogenic *Streptococcus* to escape detection by the host immune system. Cell surface-associated extracellular DNA would provide a selective advantage to *B. cereus* in its natural environment in the soil (Vilain et al., 2009). Deleting *gapB* can affect *lrgAB* expression, which is closely related to autophagy; however, it is unclear how GapB is linked to the autolysis of *B. cereus*. It is also unclear how GapB regulates programed bacterial cell lysis and releases intracellular nutrients and their DNA through the LrgAB system. We are interested in answering these questions and plan to study them in our future research.

## Conclusion

The *B. cereus* 0-9 genome contains three gap genes that encode three GAPDHs: GapA, GapB, and GapN. Only GapB plays a key role in *B. cereus* 0-9 biofilm formation. Here, we focused mainly on the function of GapB and the mechanism by which it regulates *B. cereus* 0-9 biofilm formation. GapB has GAPDH activities of both NAD^+^- and NADP^+^-dependent dehydrogenases and may play an important role in gluconeogenesis, which can control the anabolic carbon metabolism, most likely by regulating the gluconeogenesis pathway. The *gapB* gene is closely related to *B. cereus* 0-9 biofilm formation and is likely linked by gluconeogenesis. Studies on the mechanism of gluconeogenesis that affects biofilm formation have shown that the GapB is uninvolved in regulating biofilm formation through polysaccharide synthesis. The SinI/R system exists in *B. cereus* 0-9, but GapB regulates *B. cereus* 0-9 biofilm formation and does not rely on the regulatory proteins in the sinI/R system. Thus, GapB likely regulates *B. cereus* 0-9 biofilm formation relative to the release of DNA in the matrix. The transcriptome sequencing results showed that only 18 genes differed significantly after knocking out *gapB*, among which, the most significant difference between *B. cereus* 0-9 and Δ*gapB* was the *lrgAB* gene, which is annotated as an operon. We counted the viable bacteria per unit of biofilm, and the number of living cells in the biofilm formed by the Δ*gapB* strain was nearly 7.5 times than that of wild-type *B. cereus* 0-9. Therefore, we concluded that deleting *gapB* would likely to prevent autolysis of the Δ*gapB* cells, thereby reducing the release of extracellular DNA and affecting its biofilm formation. It also indicated that the GapB is located in the upstream of *lrgAB*, and it is involved in biofilm formation of *B. cereus* 0-9 by regulating the expression or activities of LrgAB. These results can help further to explain the biocontrol characteristics of *B. cereus* 0-9. We plan to conduct further research on the mechanism by which GapB regulates extracellular DNA release through the LrgAB system.

## Data Availability Statement

The datasets presented in this study can be found in online repositories. The names of the repository/repositories and accession number(s) can be found in the article/ Supplementary Material.

## Author Contributions

JZ and LM designed the experiments, carried out the experiment of the mechanism of biofilm formation, determined the dead and living cells in biofilms, drafted the manuscript, and revised the paper. YZ helped to design some experiments, carried out the experiment of quantitative detection of biofilms, and done a lot of photographing and sorting work of experimental pictures. LS constructed the expression vectors and gene knockout strains, and has done a lot of work in pioneering studies such as the phenotypic determination of mutant strains and gene expression level. QL carried out the experiment of determination of double-stranded DNAs in biofilms and collected test data. LZ and FL performed the data analyses and helped the experiment going. GW led the relevant project and helped designed the experiment. All authors contributed to the article and approved the submitted version.

### Conflict of Interest

The authors declare that the research was conducted in the absence of any commercial or financial relationships that could be construed as a potential conflict of interest.

## Supplementary Material

The Supplementary Material for this article can be found online at:

https://www.frontiersin.org/articles/10.3389/fmicb.2020.591926/full#supplementary-material

## Abbreviations

GAPDH: Glyceraldehyde-3-phosphate dehydrogenase
LB: Luria-Bertani broth
LBS: Luria-Bertani broth supplemented with some mineral salts
KBMS: King broth medium supplemented with glucose
qRT-PCR: Real-time quantitative PCR
EPS: Extracellular polysaccharide
CFU: Colony-forming units
