# Supplementary material for: GapB Is Involved in Biofilm Formation Dependent on LrgAB but Not the SinI/R System in Bacillus cereus 0-9
Source: Front Microbiol. 2020 Dec 7;11:591926. doi: 10.3389/fmicb.2020.591926 (PMC7750190; doi:10.3389/fmicb.2020.591926)
Supplement: Supplementary file 1 [file Data_Sheet_1.pdf]

"GapA"

gene complement(4883560..4884564)
/gene="gap"
/locus_tag="FRY47_25220"

CDS complement(4883560..4884564)
/gene="gap"
/locus_tag="FRY47_25220"
/inference="COORDINATES: similar to AA
sequence:RefSeq:WP_003201932.1"
/note="Derived by automated computational analysis using
gene prediction method: Protein Homology."
/codon_start=1
/transl_table=11
/product="type I glyceraldehyde-3-phosphate dehydrogenase"
/protein_id="QEF19539.1"

/translation="MTKIGINGFGRIGRNVFRAALNNSEVEVVAINDLTDAKTLAHLL
KYDTVHGTLNAEVSANENSIVVNGKEIKVIAERDPAQLPWSDYGVEVVVESTGRFTKK
SDAEKHLGGSVKKVIISAPASDEDITVVMGVNHEQYDAANHNVVSNASCTTNCLAPFA
KVLNEKFGVKRGMMTTIHSYTNDQQILDLPHKDLRRARAAAENMIPTSTGAAKAVALV
LPELKGKLNGGAVRVPTANVSLVDLVVELDKEVTVEEVNAAFKAAAEGELKGILGYSE
EPLVSIDYNGCTASSTIDALSTMVMEGNMVKVLSWYDNETGYSNRVVDLAAYMTSKGL
"

"GapB"

gene complement(4400649..4401677)
/locus_tag="FRY47_22575"

CDS complement(4400649..4401677)
/locus_tag="FRY47_22575"
/inference="COORDINATES: similar to AA
sequence:RefSeq:WP_018764454.1"
/note="Derived by automated computational analysis using
gene prediction method: Protein Homology."
/codon_start=1
/transl_table=11
/product="glyceraldehyde-3-phosphate dehydrogenase"
/protein_id="QEF19056.1"

/translation="MTRVAINGFGRIGRMVFRQAIKESAFEIVAINASYPSETLAHLI
KYDTVHGKFDGTVEAFEDHLLVDGKMIRLLNNRDPKELPWTDLGVEVVIEATGKFNSK
EKAILHVEAGAKKVILTAPGKNEDVTIVVGVNEDQLDITKHTVISNASCTTNCLAPVV
KVLDEQFGIENGLMTTVHAYTNDQKNIDNPHKDLRRARACGQSIIPTTTGAAKALAKV
LPHLNGKLHGMALRVPTPNVSLVDLVVDVKRDVTVEAINDAFKTVANGALKGIVEFSE
EPLVSIDFNTNTHSAIIDGLSTMVMGDRKVKVLAWYDNEWGYSRRVVDLVTLVVEELA
KQENVQHI"

“GapN”

gene complement(859595..861034)
/gene="gapN"
/locus_tag="FRY47_04475"

CDS complement(859595..861034)
/gene="gapN"
/locus_tag="FRY47_04475"
/EC_number="1.2.1.9"
/inference="COORDINATES: similar to AA sequence:RefSeq:WP_000213643.1"
/note="Derived by automated computational analysis using gene prediction method: Protein Homology."
/codon_start=1
/transl_table=11
/product="NADP-dependent glyceraldehyde-3-phosphate dehydrogenase"
/protein_id="QEF15645.1"
/translation="MTTSNTYKFYLNGEWRESSSGETIEIPSPYLHEVIGQVQAITRG
EVDEAIASAKEAQKSWAEASLQDRAKYLYKWADELVNMQDEIADIIMKEVGKGYKDAK
KEVVRTADFIRYTIEEALHMHGESMMGDSFPGGTKSKLAIIQRAPLGVVLAIAPFNYP
VNLSAAKLAPALIMGNAVIFKPATQGAISGIKMVEALHKAGLPKGLVNVATGRGSVIG
DYLVEHEGINMVSFTGGTNTGKHLAKKASMIPLVLELGGKDPGIVREDADLQDAANHI
ASGAFSYSGQRCTAIKRVLVHENVADELVSLLKAQVAELSVGSPEQDSTIVPLIDDKS
ADFVQGLVDDAVEKGATIVIGNKRERNLIYPTLIDHVTEEMKVAWEEPFGPILPIIRI
SSDEQAIEIANKSEFGLQASVFTKDINKAFAIANKIETGSVQINGRTERGPDHFPFIG
VKGSGMGAQGIRKSLESMTREKVTVLNLV"

“glk”

gene 1016806..1018296
/gene="glpK"
/locus_tag="FRY47_05315"

CDS 1016806..1018296
/gene="glpK"
/locus_tag="FRY47_05315"
/EC_number="2.7.1.30"
/inference="COORDINATES: similar to AA sequence:RefSeq:WP_017561267.1"
/note="Derived by automated computational analysis using gene prediction method: Protein Homology."
/codon_start=1
/transl_table=11
/product="glycerol kinase GlpK"
/protein_id="QEF15793.1"

/translation="MKKYILSLDQGTTSSRAILFNKKGEIVHSAQKEFTQHFPKPGWV

EHNAQEIWGSILAVIATCLSEADVKPEQIAGIGITNQRETTVVWDKTTSKPIYNAIVW
QSRQTAEICDELKEKGYSEMVREKTGLLIDAYFSGTKVKWILDNVEGAREKAENGDLL
FGTIDSWLVWKLSGGKAHVTDYSNASRTLMFNIHDLQWDDELLEMLTVPKSMLPEVRP
SSEIYGETIDYHFFGQNVPIAGVAGDQQAALFGQACFGEGMAKNTYGTGCFMLMNTGE
KAVASEHGLLTTIAWGIDGKVNYALEGSIFVAGSAIQWLRDGMRMFKDASESEVYASR
VESTDGVYVVPAFVGLGTPYWDSEVRGAMFGVTRGTTKEHFIRATLESLAYQTKDVLC
AMEADSGIELKTLRVDGGAVKNNFLMKFQSDILDVPVERPVINETTALGAAYLAGLAV
GYWKNQDEIKEQWHMDKRFEPTMEAKTSEELYAGWKKAIEATKAFK"

“pgm”

gene complement(4702060..4703784)
/locus_tag="FRY47_24285"
CDS complement(4702060..4703784)
/locus_tag="FRY47_24285"
/inference="COORDINATES: similar to AA
sequence:RefSeq:WP_002160034.1"
/note="Derived by automated computational analysis using
gene prediction method: Protein Homology."
/codon_start=1
/transl_table=11
/product="phospho-sugar mutase"
/protein_id="QEF19362.1"

/translation="MNWKQEFSRWLSYAQLDAELKEQLENMKQDEKKIEDSFYKNLEF
GTGGMRGELGAGTNRLNVYTVRKATKGLASFIEKLGEEAKKRGVVVAYDSRHKSPEFA
MEVAATLGARGIITYVFESLRPTPVLSFAVRHLHTVSGIVLTASHNPPEYNGYKVYGE
DGGQLPPKEADELISYVNAVEDELTVEVADVEQLKADGLLHIIGQEVDDAYAAELNNV
IINKEMVQKVGKDLKIVFTPLHGTSNISVRRGLKEVGFTDVTVVKEQELPDPNFSTVK
SPNPEEHAAFEYAIRDGEKVGADVLIATDPDADRLGVAVRNHNGEFQVLTGNQTGALM
LDYLLSQKKENGTLPENGVVLKTIVTSEIGRTIAKAYGLDTIDTLTGFKFIGEKIRQY
EESGQYEFQFGYEESYGYLIRPFCRDKDAVQSVLFACEVAAYYKSQGKTLYDGLLEVF
EKYGFFREDLVSLTLKGKDGAEKIQEMMATFRENPPKEVAGLTVVAVEDYKASIITSL
QDGHKEEIHLPKSNVLKYQLEDGSWFCLRPSGTEPKIKFYFGVKDSSLQNSEQKLLTI
KEDIMNRL"
