# Supplementary material for: GapB Is Involved in Biofilm Formation Dependent on LrgAB but Not the SinI/R System in Bacillus cereus 0-9
Source: Front Microbiol. 2020 Dec 7;11:591926. doi: 10.3389/fmicb.2020.591926 (PMC7750190; doi:10.3389/fmicb.2020.591926)
Supplement: Supplementary file 2 [file Data_Sheet_2.pdf]

Table S1 Primers used in this text.

Name	Sequence (5′-3′)	Aplication
gapB-up-*BamH*I-s	ACAC***GGATCC***TTTCGCGTTTAAACCCTCTGCA	Amplified upstream of *gapB* gene
gapB-up-*Xho*I-a	ACAC***CTCGAG***TTAGTTGTTCAAGAGTTAGCGA	
gapB-d-*Xho*I-s	ACAC***CTCGAG***TACCATTCTCCCAATACGTCCA	Amplified downstream of *gapB* gene
gapB-d-*EcoR*I-a	CACA***GAATTC***TTGGTGCATCTTTATTAGGGTA	
glk-up-*BamH*I-s	CACA***GGATCC***TCACGAAGTGTATCTACCCAAG	Amplified upstream of *glk* gene
glk-up-*XhoI-a*	CACA***CTCGAG***AAGCAATTGAAGCAACAAAAGC	
glk-d-*XhoI-s*	ACAC***CTCGAG***CGCGTGAGCTTGTTGTTCCTTG	Amplified downstream of *glk* gene
glk-d-*EcoRI-a*	ACAC***GAATTC***GATTGTGGAGGGATACTATGTC	
0-9prosinI-*Eco*RI-s	ACACGAATTCTCCCTTATTTCCTCCCCTAATA	Amplified *sinI* gene
0-9pro*sinI*-*Xho*I-a	CACACTCGAGCTATTGAGCCTGACTGGATGGC	
0-9pro*sinR*-s	ACACGAATTCTTTTTTCAATATTTAGTCATA	Amplified *sinR* gene
0-9pro*sinR*-a	CACAGGATCCCAAATGTAATTTCTCCCTAAT	
*gapB*orfcom-*BamH*I-s	CACA***GGATCC***ATGACTCGTGTGGCAATTAATGGA	Amplified *gapB* gene
*gapB*orfcom-*Xho*I-a	ACAC***CTCGAG***TTAAATGTGTTGCACATTTTCTTG	
*gapB*procom-*Mlu*I-s	ACAC***ACGCGT***CATTATTGACTAAAAAATTGTG	Amplified *gapB* gene
*gapB*procom-*Xho*I-a	ACAC***CTCGAG***TTAAATGTGTTGCACATTTTCTTG	
0-9*sinI*proorf-*Xho*I-s	CACACTCGAGCTATTGAGCCTGACTGGATGGC	Construction of *sinI* complementation vector
0-9*sinI*proorf-*Eco*RI-a	ACACGAATTCCCCTTATTTCCTCCCCTAATA	
0-9*sinR*pro-*Eco*RI-s	ACACGAATTCTTTTTTCAATATTTAGTCATA	Construction of *sinR* complementation vector
0-9*sinR*pro-*Bam*HI-a	CACAGGATCCCAAATGTAATTTCTCCCTAAT	
0-9-pMAD-chi-s	TTTACTAACAATCGCTTCAGGC	Used for verification of complementary strains
0-9-pMAD-chi-a	TTGCAAGGTTGATATTGTCCGTTA	
28a *gapB*-*BamH*I-s	ACAC***GGATCC***ATGACAACTAGCAATACGTAC	Amplified *gapB* gene
28a *gapB*-*Xho*I-a	CACA***CTCGAG***TTAAACTAAGTTTAATACAGTTAC	
lrgAB-up-BamHI-s	CACAGGATCCGTTAGCACGTGATGAACTAAAG	Amplified upstream of *lrgAB* gene
lrgAB-up-XhoI-a	CACACTCGAGGAAAATATGAATGCTTGTGATA	
lrgAB-down-XhoI-s	ACACCTCGAGTGTAACAGTAGTTGTCATCCCA	Amplified downstream of *lrgAB* gene
lrgAB-down-EcoRI-a	CACAGAATTCAAACTTTGCAGTAAAACCGACA	
gapB-Qpcr-s	GGGAGAATGGTATTTCGTCA	Quantification of

gapB-Qpcr-a	AATGCTTCTACTGTTCCGTC	*gapB* expression level
pgm-Qpcr-s	GTTGTAGCCTATGATTCTCG	Quantification of
pgm-Qpcr-a	ATTCAGGTGGATTATGGC	*pgm* expression
tasA-Qpcr-s	AATGATTTATCGGCTTGGTT	Quantification of
tasA-Qpcr-a	GTAACTTATCGCCTTGGAAT	*tasA* expression
c*alY*-Qpcr-s	CATCAGCAGCATTGGGGTTA	Quantification of
c*alY*-Qpcr-a	CTACAAGCGTCTTAGCCTTT	*calY* expression
sipW-Qpcr-s	ATCACTCACCGTATTATCG	Quantification of
sipW-Qpcr-a	CTTTCCAACTACATTTTCAG	*sipW* expression level
*16s*RNA-s	ACTGGGACTGAGACACGG	Reference genes
*16s*RNA-a	GATAACGCTTGCCACCTA	

Table S2 The expression level of the genes related to biofilm in Δ*sinR*.

The ct value	*tasA*	*sipW*	*calY*
B. cereus 0-9	22	22.5	18.6
Δ*sinR*	14.8	13.2	9.8
Δct	-7.2	-9.3	-8.8
$2^{-\Delta ct}$	147.03	630.35	445.72

Table S3. Exopolysaccharide production of cells from the biofilms of B. cereus 0-9 and ΔgapB.

	Wet weight of biofilms (g)	Blank (mg/ml)	Test (mg/ml)	EPS contents of biofilms (mg/g)
B. cereus 0-9	1.00	14.12	59.76	45.64
Δ*gapB*	0.86	10.47	50.12	46.10

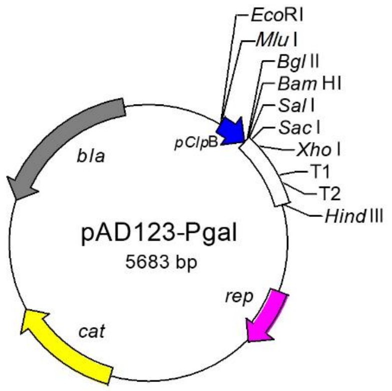

Figure S1 Structure map of pAD123-pgal vector.

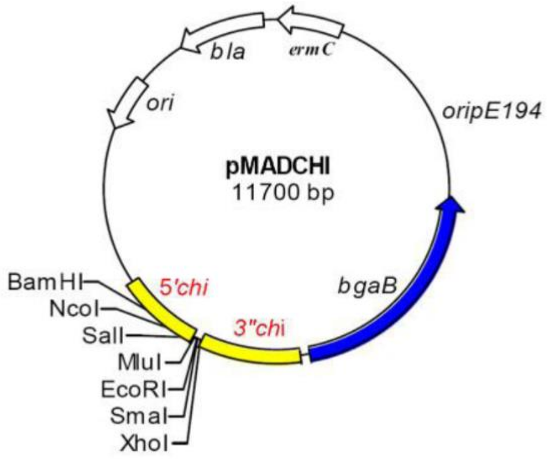

Figure S2 Structure map of pMADchi vector.

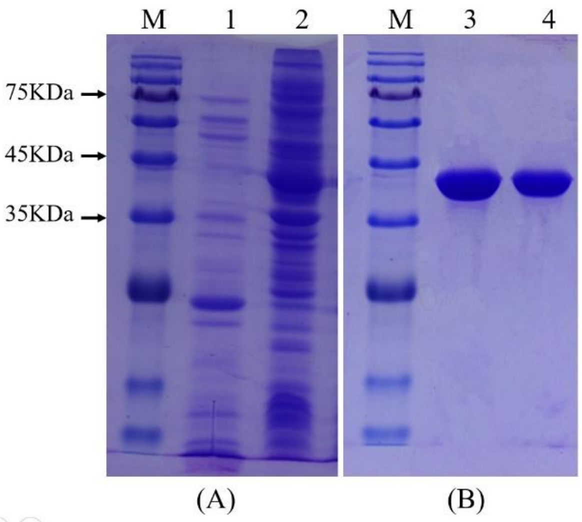

Figure S3. Identification of GapB via SDS-PAGE electrophoresis. (A) Crude proteins; (B) Purified GapB protein; M: DNA marker; Line 1: Protein expression profile of *E. coli* BL21 harboring the

pET28a plasmid; Line 2: Protein expression profiles of *E. coli* BL21 harboring the pET28a-*gapB* plasmid; Line 3: GapB purified by nickel-immobilized metal affinity chromatography; Line 4: GapB purified by dialysis.

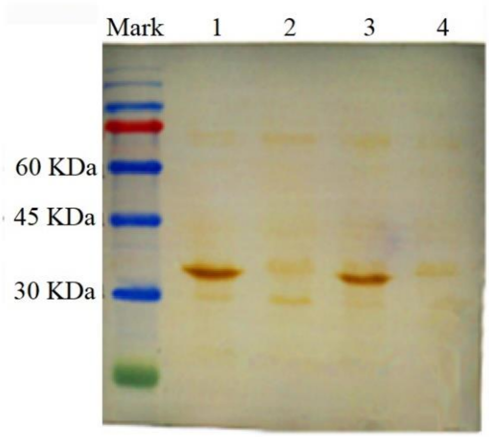

Figure S4. The expression of GapB protein detected by western blotting. Line 1: *B. cereus* 0-9; Line 2: Δ*gapB*; Line 3: Δ*sinR*; Line 4: Δ*gapB*Δ*sinR*.

Method of western blot:

The western blotting assay was performed as previously reported by Lazarev et al. (2012). The crude protein was obtained by preparation of the bacterial cell fragmentation solution, and the target protein was separated by 15% polyacrylamide gels electrophoresis according to the standard protocol. And then, protein bands were transferred onto a 0.45-µm pore-size Polyvinylidene Fluoride (PVDF) membrane (Millipore Corporation, Germany) using a Trans Blot apparatus (BioRad, Hercules, California, USA). The purified protein is sent to the company to immunize rabbits and prepare polyclonal antibodies (primary antibody). The primary antibody was incubated with target protein on the PVDF membrane, and then, secondary antibody (Goat anti Rabbit IgG antibody) was incubated with protein-primary antibody complex. In this study, we used rabbit-derived anti-GapB antibody (1:500 in Tris-buffered saline with Tween 20 [TBST]) for 1.5 h at room

temperature, followed by incubation with an alkaline phosphatase-conjugated goat anti-rabbit IgG antibody (H+L) (1:5000 in TBST, Beijing Zhongshan Biotechnology) for 1 h at room temperature. Finally, add a chemical illuminator, and the results can be observed.
